# Glasgow Royal Infirmary

**Published:** 1833-07

**Authors:** 


### GLASGOW ROYAL INFIRMARY.

### *Carcinoma of the Rectum; Operation; Recovery.*

Alexander Campbell, aged twenty-eight, admitted 14th November, 1832. Extending for nearly an inch around verge of anus is a circle of unhealthy looking ulceration, having high everted edges, and a rough, callous surface, discharging copiously a thin unhealthy matter. On going to stool he experiences great pain, and there is occasionally a slight discharge of blood; and, when the fæces are liquid, they frequently pass out involuntarily. General health seems somewhat impaired. Complaint is of a year's duration, and began with what seems to have been external hemorrhoidal tumors, which were excised by a surgeon. The cut surfaces, however, did not heal, and sore has gradually attained its present appearance. A suppository of hemlock and opium to rectum night and morning. Solution of chlorine to external ulcer.

9th December. Suppository produced considerable relief from pain, but the appearance of the external sore has been little altered by the lotion. No improvement in general health. Countenance sallow, lips palid, pulse 106, weak. By advice of consultation, the following operation was performed for removal of diseased parts.

The patient being placed in the ordinary posture for lithotomy, and the urine having been previously discharged, a sound was introduced into bladder to elevate the prostate gland. An incision embracing diseased parts, and extending through skin and considerable part of cellular substance, was then made. Anteriorly the gut was separated from its surrounding connexions by cautious dissection; laterally and posteriorly the knife was passed up the depth of the disease, and its point made to penetrate the walls of the rectum. It was then carried round a considerable portion of the circumference of the gut, guided by the finger introduced into the rectum. The greater portion of the disease being thus removed, it was found that several parts of diseased intestine still remained behind. These were subsequently dissected out along with several glands, which lay internal to the skin, and were enlarged and indurated. Their cellular connexions being pretty strong, they were removed by the finger with some difficulty. Still more difficulty was found in removing some which were firmly adherent to the right lateral lobe of prostate gland. The diseased parts were in this manner removed as far as could be ascertained. The interior of the rectum felt smooth, and its walls soft and pliable. A considerable quantity of blood was lost during the operation; and this escaped from an infinity of small vessels, the greater number of which speedily retracted, a single vessel only requiring a ligature. At one time during the operation there was an approach to syncope, but the pulse never disappeared at the wrist. The cavity formed by excision of diseased parts was filled by several pieces of sponge, and a compress applied externally, supported by a T bandage.

A short time after removal to bed, says he feels little pain, and his sensations do not differ in a material degree from what they were previous to operation. Pulse 116, rather feeble.

10th. Was restless during the night, and had great thirst. Fluid taken into stomach immediately rejected by vomiting. These symptoms were relieved by effervescing draughts, and the application of turpentine to region of stomach. At present skin is hot, but has had no rigor. Is free from abdominal pain, and can bear any degree of external pressure. Thirst less urgent. No nausea nor tendency to vomit. Has passed urine freely, and without pain. Pulse 144, rather sharp. On removing dressings, sponge found to have distended the wound, being every where firmly adherent to its surface. Slight pain, but character of pain changed. No surrounding inflammation. No stool. Half an ounce castor oil immediately. Thirty drops vin. antim every third hour. Oiled lint to wound.

12th. Size of wound appears to diminish, and its surface is coated over with a lymphatic exudation. No redness nor swelling of surrounding integuments. Says he feels much relief from pain since operation. No abdominal uneasiness, and no return of vomiting. Discharges urine freely. One copious evacuation from bowels without pain. Skin cool. Tongue clean and moist. No thirst. Pulse 128, rather feeble. Appetite deficient. ℞. Sulph. Quiniæ ʒss. Acid., Sulph. Aromat. ʒij., Tinct. Gent. comp. ℥ij. Aq. Pur. ℥viii. M. fiat mistur. cujus sum. coch. max. 4ta qq. horâ.

14th. No pain in wound, and no surrounding inflammation. Improves somewhat in strength. Bowels regular. Pulse 118. A beef-steak and pint of porter daily.

22d. Strength recruits. Sleeps well. Countenance cheerful. Is free from pain. External wound diminishes, and denuded parts begin to be covered with a thin pellicle of skin. Is still unable to retain his fæces. Pulse 108, of moderate strength.

During the month of January he continued to improve, and the wound went on contracting. It was necessary to introduce a bougie frequently to keep the rectum patent, there being a disposition in the parts to contract more than was desirable. Early in February, without any evident cause, he had an attack of abdominal irritation, preceded by rigors, and accompanied with smart pyrexia and considerable pain of hypogastric region. These symptoms required leeches, laxatives, and effervescing draughts, by which they were subdued. The wound is now nearly healed, but the bougie has still to be employed, to prevent too much contraction. The fæces can be retained, except when they are very fluid, in which case a small quantity sometimes escapes involuntarily. His general health is now tolerably good, and I expect to dismiss him in a short time, cured.—*Glasgow Medical Journal.*

